# Vat Photopolymerization of Glycerol Carbonate Methyl Itaconate for Sustainable 3D Printing

**DOI:** 10.1021/acssuschemeng.5c02799

**Published:** 2025-07-29

**Authors:** Rosario Carmenini, Matteo Dalle Donne, Erica Locatelli, Letizia Sambri, Loris Giorgini, Laura Mazzocchetti, Mauro Comes Franchini

**Affiliations:** Department of Industrial Chemistry “Toso Montanari”, 9296 University of Bologna, Bologna 40136, Italy

**Keywords:** additive manufacturing, biobased materials, itaconic acid, vat photopolymerization, flame retardant

## Abstract

The growing interest in 3D printing highlights the need for new formulations that enhance sustainability by utilizing renewable substrates due to their predominantly fossil-derived nature found in most commercial applications produced. In this context, itaconic acid and glycerol carbonate (a glycerol derivative) are two promising building blocks that can be combined to synthesize a cyclic itaconate carbonate suitable for vat photopolymerization. This study describes the synthesis and chemical characterization of a novel monofunctional photopolymerizable monomer, glycerol carbonate methyl itaconate (GCI), which was incorporated into two resin types, with soft and rigid properties, demonstrating high compatibility and increasing the overall biobased content up to 77 wt %. Mechanical testing revealed that the GCI enhanced the rigidity of soft resins while improving the elongation properties of rigid formulations. These results indicate that GCI not only improves the sustainability of fossil-based resins but also imparts added functionality, broadening the potential applications of itaconic acid–derived monomers.

## Graphical abstract

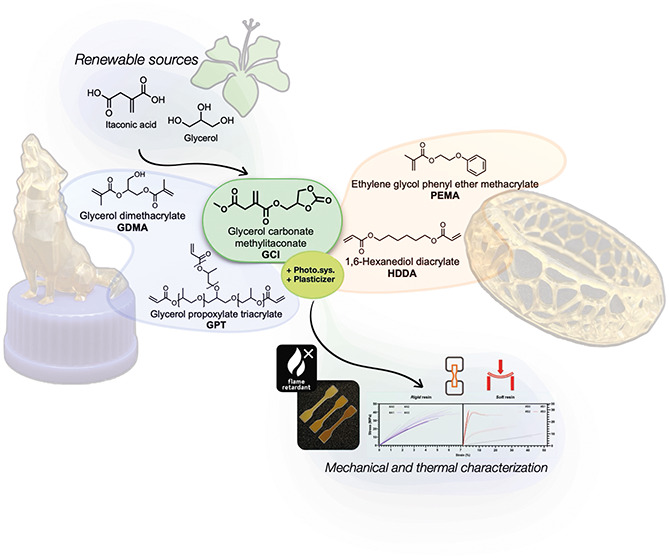

## Introduction

Additive manufacturing is gaining popularity in fields such as medicine, engineering, fashion, construction, and food owing to its ability to create complex designs with high resolution and enhanced customization possibilities. This sector reached USD 18 billion in 2022, highlighting its impressive growth potential.^1^ Vat photopolymerization is a cutting-edge technology that employs a light source to cure liquid resin, creating CAD-designed objects layer by layer with micrometer precision. While 3D printing is naturally less environmentally harmful owing to its lower material consumption compared to traditional techniques, most commercial monomers used in resins are still fossil-based, such as acrylate and methacrylate molecules. Therefore, to minimize CO_2_ emissions, it is increasingly crucial to integrate natural monomers or polymers into vat photopolymerization resins.^2,3^ A collection of recent studies has identified biobased alternatives to fossil-based monomers by utilizing various natural building blocks, including lignin derivatives,^4,5^ vegetable oils,^6^ functionalized natural polymers,^7,8^ and therpene.^9,10^ These alternatives have shown remarkable results in terms of mechanical performance and more environmentally friendly synthetic strategies. One promising renewable building block is itaconic acid (methylenesuccinic acid), which can be produced by fermenting sugars or complex carbon sources such as starch and molasses using *Aspergillus terreu*s strains. A green strategy involves leveraging the photopolymerizable activated double bonds of the itaconic backbone,^11,12^ together with its functionalization using another well-known renewable building block, propane-1,2,3-triol (glycerol). Glycerol is a globally recognized renewable resource that can be incorporated into various sustainable materials and is already employed in industries such as cosmetics, automotive, and food.^13^ It can be synthesized either through the transesterification of triglycerides or via anaerobic fermentation of sugars mediated by *Saccharomyces cerevisiae*.^14^ Glycerol carbonate (GC), together with itaconic acid derived from renewable sources such as glycerol, represents a sustainable solution for 3D printing by reducing the dependence on petrochemical materials. Its cyclic carbonate group enables chemical interactions with other polar groups present in the monomer mixture, expanding the formulation possibilities of the resins. Various syntheses of GC are available in the literature, involving substrates such as organic carbonates^15^ or urea. Although urea is mainly produced via the Haber–Bosch process, many studies propose its green synthesis through CO_2_ capture and the use of green hydrogen.^16^ This provides an excellent starting point for the synthesis of GC because urea can react with glycerol in the presence of a Lewis acid without solvents. To the best of our knowledge, GC has only been used in vat photopolymerization by Schimpf et al.,^17^ alongside a methacrylic component.

Herein, we aimed to use GC while replacing fossil-based methacrylates with more environmentally sustainable itaconic acid. Therefore, itaconic acid- and glycerol-based derivatives were used to synthesize a new fully renewable monomer for vat photopolymerization: glycerol carbonate methyl itaconate (GCI). Interestingly, combining itaconic acid monomethyl ester with GC creates a liquid monomer suitable for 3D printing, which is a fundamental characteristic of the final formulation that can be printed. Notably, despite its lower reactivity compared to conventional fossil-based acrylates and methacrylates,^18^ itaconic acid demonstrates excellent mixability in the final resin. The resulting liquid monomer, GCI, was easily and successfully integrated into resin formulations with both hard and soft properties in printed objects. This demonstrated its high versatility and compatibility with other monomers, yielding high-resolution final artifacts. The biobased content was calculated, and the mechanical properties, including tensile and bending strength and hardness, of the printed objects were analyzed. GCI not only maintained high resolution in the final artifacts but also substantially enhanced the mechanical properties of the formulated resins, expanding their potential applications beyond the initial sets of resins.

## Experimental Section

### Materials

All chemicals were purchased from Sigma-Aldrich (St. Louis, MO) and used as received. Chloroform was dried by distillation over CaCl_2_ and used promptly. Grindsted Soft-N-Safe (9-hydroxystearic acid monoglyceride triacetate, SNS) was purchased from Danisco (Brabrand, Denmark).

### Synthesis of Glycerol Carbonate (GC)

In a 1 L double-neck round-bottomed flask equipped with a large magnetic stirrer, 146.2 mL (2 mol) of glycerol was added and heated to 60 °C. Then, 121.2 g (2 mol) of urea was inserted portioning to obtain a homogeneous system. Finally, 5.45 g (0.04 mol) of ZnCl_2_ was inserted, and the mixture was kept under a vacuum (21 mbar) at 150 °C for 2 h to ensure the reaction’s completeness. Then, the product was cooled to room temperature, isopropanol was added to remove the catalyst residues and the mixture was filtrated. The solvent was evaporated, and a high vacuum distillation was performed at 200 °C distilling the glycerol. Then, the GC present in the distillation flask was washed with acetonitrile and ethyl acetate, and all of the solvents were evaporated obtaining a viscous yellow liquid. ^1^H NMR (600 MHz, CD_3_CN) δ 4.76–4.74 (m, 1H), 4.47 (t, 1H), 4.30 (dd, 1H), 3.79–3.76 (m, 1H), 3.60–3.58 (m, 1H), 3.32 (t, 1H) ^13^C NMR (600 MHz, CD_3_CN)­δ 156.42, 77.83, 66.78, 61.94; ESI-MS = 117 [M – H]. Yield = 55%.

### Synthesis of Monomethyl Itaconoyl Chloride

In a 1 L round-bottomed flask under a nitrogen atmosphere equipped with a CaCl_2_ drying tube, 180 mL of oxalyl chloride (2.1 mol) was added to itaconic acid monomethyl ester (216 g, 1.5 mol). The reaction was allowed to take place by stirring overnight at room temperature. Once the reaction was completed, unreacted oxalyl chloride was distilled at room temperature under a high vacuum and recovered for further use. Then, the temperature was increased to 120 °C, and pure monomethyl itaconoyl chloride was distilled off as a colorless liquid from the reaction mixture and stored under an inert atmosphere in the dark at −20 °C. ^1^H NMR (400 MHz, CDCl_3_) δ 6.72 (s, 1H), 6.18 (s, 1H), 3.72 (s, 3H), 3.40 (s, 2H). Yield = 86%.

### Synthesis of Glycerol Carbonate Methyl Itaconate (GCI)

In a three-neck dry 500 mL flask equipped with a large magnetic stirrer and a rubber septum, under a nitrogen flow, dry chloroform (100 mL) was added followed by 21.9 mL (0.26 mol) of glycerol carbonate and 43.8 mL (0.31 mol) of dry triethylamine, and then the mixture is cooled down to 0 °C using an ice bath. When the solution is cooled, 39 mL (0.31 mol) of itaconoyl monochloride is added dropwise over 1 h by using a drop funnel. When the addition is completed, the ice bath is removed, and the mixture is left under stirring and nitrogen atmosphere for 2h. The product contained in the organic solvent was filtered using Celite and washed several times with water and brine. Then, the solvent was dried over Na_2_SO_4_ and evaporated under a high vacuum pump to afford glycerol carbonate methyl itaconate as an orange viscous liquid. ^1^H NMR (600 MHz, C CDCl_3_) δ 6.36 (s, 1H), 5.78 (s, 1H), 4.96 (m, 1H), 4.56 (t, 1H), 4.37 (m, 3H), 3.69 (s, 3H), 3.35 (s, 2H); ^13^C NMR (600 MHz, CDCl_3_) δ 171.03, 165.56, 154.50, 132.83, 130.35, 73.84, 66.03, 63.66, 52.22, 37.40; ESI-MS = 244 [M + Na+]. Yield = 92%.

### Formulation of Photocurable Resins

Before printing, all of the formulations were mixed with a fixed-speed planetary mixer (Precifluid P-MIX100) for 4 min and the mixtures were poured into the printing plate using glycerol dimethacrylate (GDMA), glycerol propoxylate triacrylate (GPT, average Mn = 428), ethylene glycol phenyl ether acrylate (PEMA), 1,6-hexanediol diacrylate (HDODA), and GCI as monomers (Table S3). To all formulations were also added ethyl (2,4,6-trimethyl benzoyl) phenyl phosphinate (Et-APO, 2.0 wt %) as the photo radical polymerization initiator, 2-isopropyl thioxanthone (ITX, 0.3 wt %) as the photoabsorber, and 2,6-di*tert*-butyl-4-methylphenol (BHT, 0.5 wt %) as the radical trapping stabilizer, while Grindsted Soft-N-Safe (9-hydroxystearic acid monoglyceride triacetate, SNS, 7.2%) was added as the plasticizer. To all of the specimens has been added a small amount of natural dye (purpurin 0.005 wt %) to demonstrate the capability of the final resin sets to be colored, expanding the applicability fields. Only the two high content of GCI (#A3 and #B3) have a different amount of photopolymerization system (Et-APO: 3.0 wt %; ITX, 0.3 wt %; BHT, 0.7 wt %; SNS, 6 wt %) and plasticizer since the specimens were not complete at the end of the 3D printing as shown in Table S3.

### Stereolithographic 3D Printing Photocurable Resins

The dog bones for the tensile test have been designed using computer-assisted design (CAD) following the ISO-37 Type 2 specifications (5 × 2 mm^2^ cross-section, 25 mm gauge length). Additionally, rectangular bars measuring 100 × 40 × 10 mm^3^ were designed for three-point bending tests according to ISO 178. The 3D models were sliced by using Chitubox V 1.7.0 software. First, the.stl files corresponding to the tensile and bending test specimens were imported onto the virtual plate, and then the printing parameters (such as layer height, exposure time, and lifting speed) were configured before slicing into the corresponding g-code. Layer height for all specimens was 0.1 mm, and the irradiation time per layer was optimized for each formulation, ranging from 80 to 120 s. Generally, the printing time increases with the percentage of synthetic monomers. The g-code files were exported, and all of the samples were printed using the Phrozen Sonic 4K 3D printer equipped with a 6.1 in. 50 W monochrome 405 nm, ParaLED Matrix 3 UV screen (3840 × 2160 resolution, 4K), and the formulated resins were poured into its vat. After the printing, the samples were washed with isopropanol to remove the nonpolymerized resin, detached from the plate, and dried for 2 h hours at room temperature. Then, 3D-printed objects were postcured at 25 °C for 10 min in a UV curing oven (Sharebot CURE, λ = 375–470 nm, 120 W) and exposed to air and environmental light conditions for 1 week before mechanical testing. The potential effect of the washing step on the mechanical properties of the photocured materials was assessed by comparing the mechanical properties of the washed and unwashed samples, and no appreciable differences were detected.

### Chemical and Mechanical Characterization

^1^H and ^13^C NMR spectra were obtained on a Varian Inova (14.09 T, 600 MHz) and a Varian Mercury (9.39 T, 400 MHz) NMR spectrometers. In all recorded spectra, chemical shifts have been reported in parts per million of frequency relative to the residual solvent signals for both nuclei (^1^H: 7.26 ppm and ^13^C: 77.16 ppm for CDCl_3_, ^1^H: 1.94 ppm and ^13^C: 1.32, 118.26 ppm for CD_3_CN). ^13^C NMR analysis was performed using the ^1^H broadband decoupling mode. Mass spectra were recorded on a micro mass LCT spectrometer using electrospray (ES) ionization techniques. ATR-FTIR analysis has been performed using a Cary 630 FTIR spectrometer. Agilent Rotational viscosity measurements were performed on an Anton Paar Rheometer MCR102 with a cone–plate CP50–1 configuration (1° angle and 25 mm diameter). The experiments were achieved with a constant rotational frequency of 1 Hz in the temperature range +10/+40 °C and a heating rate of 5 °C/min. A Remet TC10 universal testing machine was used to perform all of the tensile and flexural tests. The instrument was equipped with a 10 N cell, with a crosshead speed of 1 mm/min for both tests, according to the ISO 37 Type 2 (5 × 2 mm^2^ of section, 25 mm gauge length) and ISO 178 (100 × 40 × 10 mm^3^ rectangular bars) specifications. Hardness was evaluated using an analogous Shore D durometer (Remet). DSC curves were recorded on a TA Instruments Q2000 under a nitrogen atmosphere with a flowing rate of 50 mL min^–1^. Each sample was weighed and sealed in an aluminum crucible and heated from room temperature to 250 °C at a heating rate of 20 °C/min.

Thermogravimetric analyses (TGA) were performed on a TA Discovery Q500 TGA Instruments under a nitrogen atmosphere (40 mL/min) and air (40 mL/min) at a heating rate of 20 °C/min.

## Results and Discussion

### Synthetical Route for Glycerol Carbonate Methyl Itaconate

The synthesis of GCI involves the reaction of glycerol and urea (Figure 1). Various methods exist for producing cyclic carbonate GC from these two biobased building blocks. This study builds on the work of Park et al.^19^ using a catalytic amount (2 mol %) of a Lewis acid (ZnCl_2_). The choice of a zinc-based catalyst is owing to its lower toxicity and cost compared to other metal-based Lewis acids.^20^

**Figure 1:**
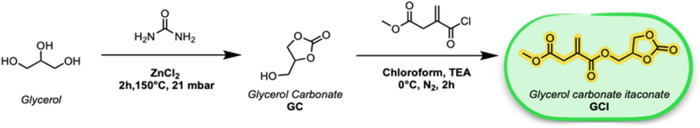
Synthesis of GCI from glycerol and urea and the following reaction with monomethyl itaconoyl chloride to produce biobased GCI monomer.

The initial reaction involves a nucleophilic attack by the two primary glycerol hydroxyl groups on urea, forming a carbamate intermediate, which then converts to carbonate with the elimination of two ammonia molecules.^21^ Optimizing the reaction conditions was crucial for enhancing the yield and purity of the GC synthesis. Park et al. showed that the use of ZnCl_2_ as a Lewis acid at 150 °C for 2 h maximizes glycerol conversion to the carbonate. However, exceeding this temperature can cause urea decomposition. Our screening of molar ratios between glycerol and urea (1:1, 0.9:1, and 1:2) found that the equimolar path resulted in the highest yield (81%), compared to 62 and 34% for the other two. Yield decreased with increased urea amounts, leading to additional byproducts. To shift the reaction equilibrium toward the product and efficiently collect the ammonia salt coproduct, a vacuum was applied to the reaction system.^19^ The crude product of the reaction was purified by distilling glycerol residues, recovering GC in the distillation flask, and washing with acetonitrile/ethyl acetate. This process yielded a high-purity product with a total yield of 55% after purification. The product was confirmed via electrospray ionization mass spectrometry (ESI-MS, Figure S4), infrared (IR, Figure 2b), and nuclear magnetic resonance (NMR, Figure S1–3) spectroscopy. ^1^H-, ^13^C, ^1^H–^1^H–COSY, and ^1^H–^13^C-HSQC NMR spectroscopy showed differences in hydrogen coupling in the carbonate ring and the hydrogen of the tertiary carbon and the coupling between the OH group and the two hydrogens of the lateral chain, showing a triplet (3.3 ppm; Figure 2a). Furthermore, attenuated total reflectance Fourier transform infrared (ATR-FTIR) spectroscopy confirmed the presence of the following main functional groups in the product: O–H stretching at 3400 cm^–1^ shifted and decreased in intensity compared to glycerol; carbamate C=O stretching at 1755 cm^–1^; and C–C and C–O stretching of the 2-hydroxyethyl chain at 1200–1000 cm^–1^, supporting the presence of the product: GC^22^ (Figure 2c). GCI was obtained via the acylation of the synthesized GC with monomethyl itaconoyl chloride, producing the chlorination of itaconic acid monomethyl ester, as recently reported.^23^ The obtained monomethyl itaconoyl chloride was purified using distillation, yielding the high-purity material. This was then added dropwise to a solution of GC and triethylamine to trap HCl produced during the nucleophilic attack by the primary hydroxy group, ensuring the solvent was anhydrous to prevent the undesired hydrolysis of the acyl chloride previously synthesized. The esterification reaction occurred instantaneously between itaconic acid chloride and glycerol. The structure of the desired GCI product was confirmed via ESI-MS and NMR spectroscopy (Figures S8 and 2b/S5–S7). “The same reaction as glycerol carbonate can be successfully carried out through monomethyl itaconate using dibutyl tin oxide distilling the final product, although in lower yields (see Supporting Information).”

**Figure 2:**
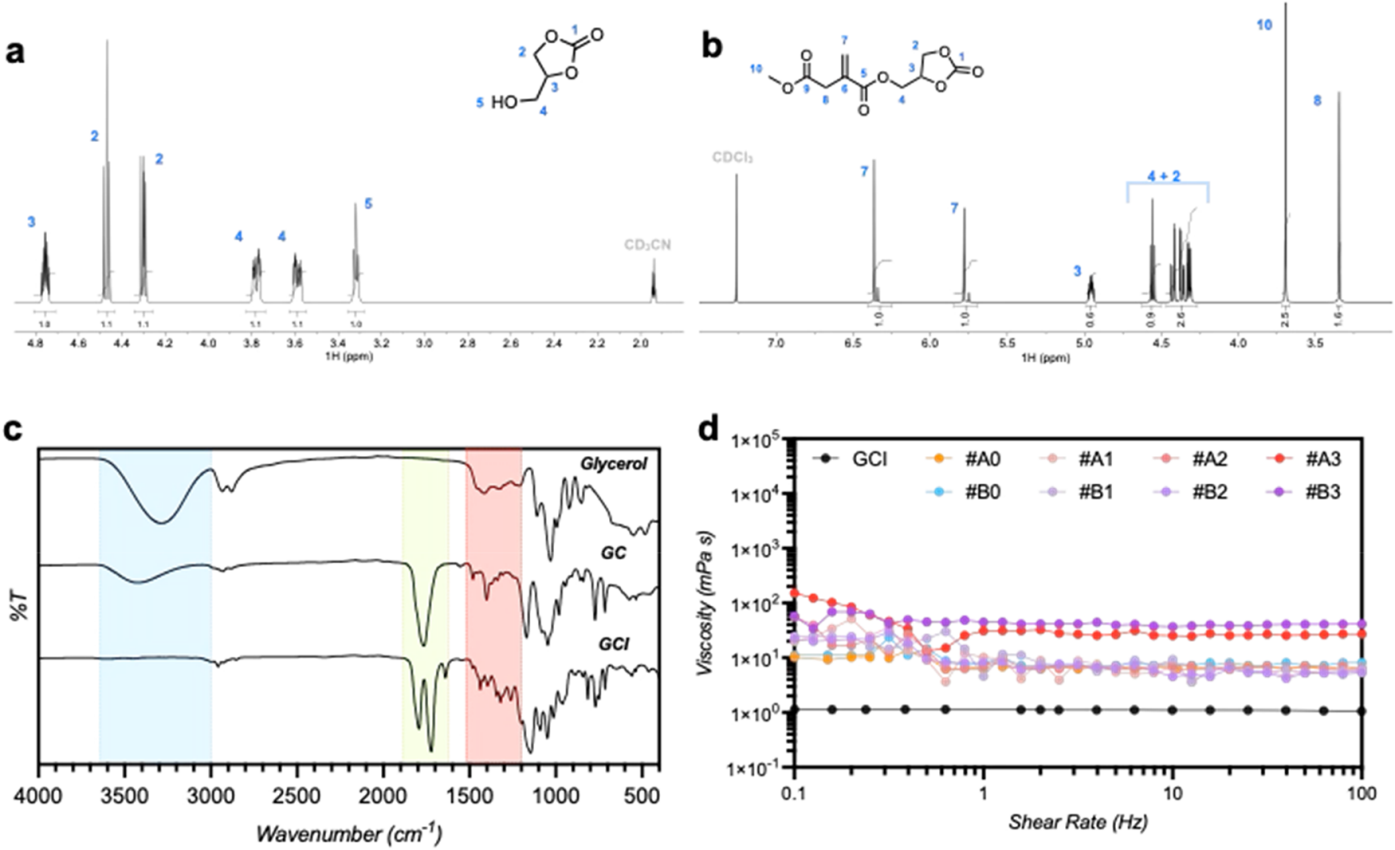
^1^H NMR (CD_3_CN and CDCl_3_, 600 MHz) analysis of glycerol carbonate (a) and glycerol carbonate methyl itaconate [GCI] (b) with the corresponding signal attributions. For GCI only one isomer is reported but the attribution is of all isomers formed. (c) Spectroscopic characterization of glycerol, glycerol carbonate [GC], and glycerol carbonate methyl itaconate [GCI] with the most important wavenumber zone highlighted of the −OH stretch, C=O stretch, and C–C/C-O stretch. (d) Rheological analysis of the GCI monomer and all resin sets: viscosity (mPa·s) as a function of shear rate at constant temperature (27 °C).

Monomethyl itaconoyl chloride, prepared using this method, consists of a mixture of 95% α, β-unsaturated acyl chloride and 5% α,β-unsaturated methyl ester,^24^ making it plausible to observe the formation of two isomeric products. In fact, the ^1^H NMR spectrum of GCI (Figure 2b) shows two pairs of signals related to the vinylic groups. The formation of the desired product is further supported by comparing the ATR-IR spectra (Figure 2c) of the reagents and GCI; the disappearance of the −OH stretch at 3000–3500 cm^–1^ and the presence of two C=O stretches at 1716 and 1788 cm^–1^ confirm the presence of the expected product.

To evaluate the suitability of GCI for photopolymerization resins, we conducted rheological analyses. The results, shown in Figures 2d and S12, demonstrate that the monomer and the final resins set behave as a Newtonian fluid with its viscosity remaining constant as the shear rate increases and a linear correlation between shear stress and shear rate. This property is crucial for photopolymerization monomers, as it ensures the consistent viscosity of the material under stress during loading or printing, thereby maintaining the quality of the printing process. Additionally, the viscosity decreases with increasing temperature, a common behavior for 3D printing monomers. Nevertheless, the viscosity values are comparable to those of industrial monomers, such as poly­(ethylene glycol) diacrylate. Typically, resins with low viscosity are preferred for improved flowability, facilitating the efficient filling of new layers during printing.^1^

### Formulation of Photocurable Resins and Stereolithographic 3D Printing

The prepared GCI was formulated with commercial photocurable monomers to create resins for vat photopolymerization, as shown in Figure 3. This was performed to assess the impact of our new biobased monomer on the mechanical properties by combining it with fossil-based (meth)­acrylates and acrylates. By changing the nature of the fossil-based monomers and the number of double bonds in the mixture with the biobased monomer, two different sets of resins (#A and #B) were created, resulting in eight different formulations categorized into rigid and soft resins. The synthesized photocurable monomers were mixed in a fixed-speed planetary mixer for 4 min, as per the compositions listed in Table S3, and then poured into the 3D printer vat for spatially controlled photopolymerization. For all formulations, the monomer percentages (with a total sum of 100%) were adjusted with 10 wt % of the photoinitiation system and plasticizer, as detailed in Table S3.

**Figure 3:**
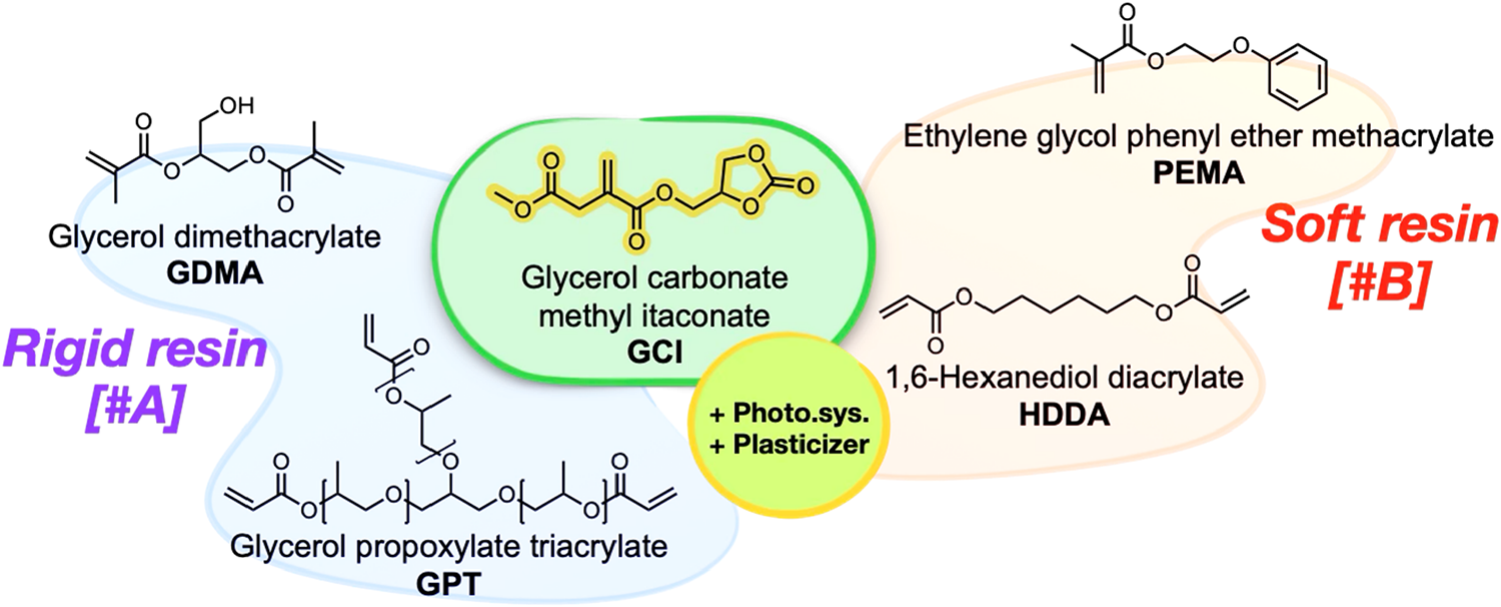
Schematic representation of the two resin sets [#A and #B] each formulated combining the fossil-based monomers with the biobased GCI, the photoinitiator system [Et-APO, BHT, ITX], and plasticizer [Grindsted Soft-N-Safe].

A first set of resins was prepared by mixing GCI with a 1:1 mixture of glycerol dimethacrylate (GDMA, mixture of isomers) and glycerol propoxylate triacrylate (GPT, average Mn = 428). In this rigid formulation, the GCI content was gradually increased to 70 wt %, the printing limit incorporating a high percentage of biobased monomer. The second set of resins was prepared by mixing GCI with a 7:3 mixture of ethylene glycol phenyl ether methacrylate (PEMA) and 1,6-hexanediol diacrylate (HDDA). The maximum GCI content in both formulations (#A, and #B) (70 wt %) was determined through screening before mechanical testing, as higher concentrations than this cause specimens to detach from the plate, especially bending samples. For both series, a blank test was conducted: (#A0) with only GDMA and GPT (in a 1:1 ratio) and (#B0) with only PEMA and HDDA (in a 7:3 ratio). Three tests were performed, progressively increasing the concentration of biobased monomer for the rigid resin (#A1, #A2, and #A3) and soft resin (#B1, #B2, and #B3), as outlined in Table S3. All formulations also included a photopolymerization system and plasticizer.

The biobased content of the eight formulations was determined based on the renewable and sustainable building blocks used for each component as well as the overall biobased mass content of each formulation. Itaconic acid, urea, and glycerol derivatives, including GDMA and GPT, are sustainable molecules, as explained in the Introduction section. For other photocurable monomers, 1,6-hexanediol in HDODA and ethylene glycol inside PEMA are fully biobased, derived from biomass-derived HMF^25^ and various biobased biomass-derived feedstocks such as ethanol or sorbitol, respectively.^26^ However, the biobased content of the photoinitiating system is 0 wt %, while the plasticizer Grindsted SNS is 100 wt % biobased. The most biobased resins for the rigid and soft sets were #A1 and #B3, with biobased contents of 76 and 77 wt %, respectively, where GCI is present in the maximum content. All resins were successfully 3D-printed with high spatial accuracy and resolution and allowed the addition of small amounts of a natural dye (purpurin, 0.005 wt %) to create colored 3D objects (Figure 4b, d). Furthermore, each formulation was used to print tensile and flexural test specimens, which were then tested according to the ISO 37 Type 2 and ISO 178 specifications, respectively. Owing to the less reactive nature of itaconic acid residues compared to acrylates and methacrylates, radical polymerization required adjustments in the instrumental printing parameters. In our case, the LCD 3D-printing technology limited the parameters we could explore to the exposure time per layer, which was extended up to 2 min for every 0.1 mm of object height (Table 2). All printing parameters have been optimized, beginning with a minimum time of 25 s per layer. The layer exposure was then increased until 2 min per layer while maintaining a consistent photoinitiating system across all #AX and #BX formulations (2 wt % Et-APO, 0.3 wt % THX, and 0.5 wt % BHT); except, for #A3 and #B3 formulations, the amount of the photoinitiating system was increased to 3 wt % Et-APO, 0.3 wt % THX, and 0.7 wt % BHT. In all of the resin sets has been added 7 wt % of plasticizers (Grindsted Soft-N-Safe). However, highly advanced systems could optimize additional parameters, such as light power, to reduce the 3D-printing process time.

**Figure 4:**
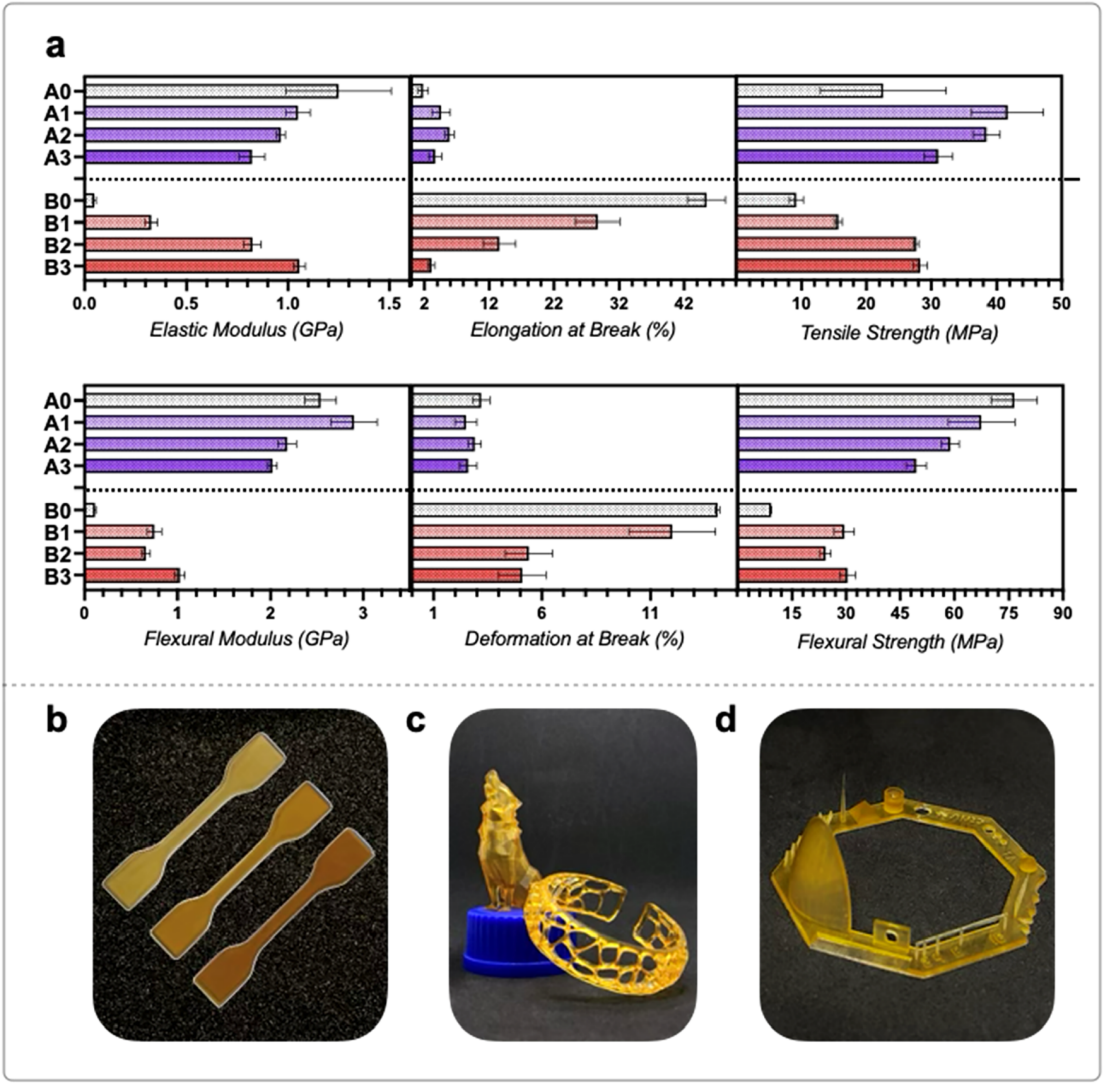
(a) Graphical comparison of mechanical properties of the eight different formulations divided into rigid (#AX, purple) and soft (#BX, red). The “blank” (without the biobased monomer) is implemented in #X0 formulations for each set. Digital camera picture of high-resolution 3D-printed objects. (b) Tensile test specimens obtained by printing resins A1, A2, and A3 (from right to left); (c) high-resolution objects printed with the #A3 (wolf) and #B2 (bracelet) resins; (d) 3D test model printed using #A3 resin with 70 wt % of GCI.

### Thermal and Mechanical Properties of 3D-Printed Materials

Thermal and thermo-oxidative stabilities of the different resins were characterized via thermogravimetric analysis (TGA) under nitrogen and air, with the results presented in Table 1. Under an inert atmosphere, such as nitrogen or argon, only thermal degradation from the breaking of chemical bonds occurs. In air, both oxidation and thermal decomposition are observed. The results showed a decrease in thermal stability in samples with higher GCI content compared to the GCI-free resins. The most noticeable difference detected in *T*_onset_, evaluated by examining the onset of the first prominent weight loss in the TGA curves, was observed between samples #A0 and #A1, with a drop of 36 wt %. Notably, the addition of 50 or 70 wt % GCI resulted in nearly identical thermal stability values, suggesting that, as expected, the presence of carbonate-containing units alters overall thermal stability without compromising the performance, at least under the conditions typically used for 3D-printing processing and part exploitation, which are well below the glass transition temperature (Tg). In the present case, the degradation temperature is still remarkably high and does not negatively affect the usability of such compounds even in critical and requiring applications: hence, the use of GCI while demoting slightly *T*_onset_ is hampering potential applications.

**Table 1:** Thermal Properties Based on TGA (in Inert and Oxidative Atmosphere) and DSC Measurement of the Eight Different Formulations Divided into Two Sets: Rigid (#AX) and Soft (#BX)

	TGA in nitrogen		TGA in air		DSC
#	*T*_Onset_ (°C)	residual fraction (%wt.)	*T*_Onset_ (°C)	residual fraction (%wt.)	*T*_g_ (°C)
A0	387	2.8	130	0	54
A1	246	5.2	346	0	58
A2	243	6.7	241	0	46
A3	242	10	241	0	49
B0	416	2.2	409	0	28
B1	248	5.0	347	0	61
B2	238	7.0	238	0	57
B3	239	10	238	0	57

In the TGA curves (Figure S13) under an air atmosphere, two main thermal degradation stages were observed. The first stage is the degradation of the cross-linked network that showed a sharp drop in thermal stability, with a weight loss onset of ∼ 130 °C, while the second stage, absent in the thermograms recorded in an inert atmosphere, is ascribed to the thermal oxidation of the formed char.

A similar trend to that observed for rigid system (A) for TGA run in inert condition whose also detected in the soft one (B), with a drop in weight loss between #B0 and #B1of about 40%, and a flattening of such a trend for higher GCI content, namely 50–70 wt %. Indeed, concerning the weight drop, the increasing GCI percentage in the samples led also to an enhanced residual mass, which was comparable between the two resin systems (A and B) and always markedly higher than that of the GCI-free formulation. The high glycerol content resulted in an increased carbonaceous residue at the end of the analysis. This behavior in glycerol-based organic structures has been previously observed, and glycerol derivatives are often used as flame retardants, as their charring ability is a known indicator of the fire resistance of a material.^27,28^ Results in Table 1, however, showed different thermo-oxidative trends for stability values for Resin B. Indeed, the soft resin (B) exhibited thermal stability comparable to that recorded under inert conditions. Samples loaded with 30 wt % GCI (#A1 and #B1) showed greater thermo-oxidative stability compared to those tested in nitrogen, while samples loaded with 50 and 70 wt % carbonate-based units displayed comparable values for both thermal and thermo-oxidative stability.^29^

Differential scanning calorimetry (DSC) curves of resin system A with varying GCI percentages are shown in Figure S11, with #A1 thermogram displaying a significant exothermic peak, which is likely associated with an incomplete residual cross-linking process. This exothermic behavior, however, is significantly lowered, albeit still being slightly visible (See Figure SI1 in SI) in the other samples, confirming a decrease in unreacted double bonds. Additionally, when examining the *T*_g_, a nearly constant relaxation temperature is observed when comparing #A1 with #A2 and #A3, showing no prominent self-plasticizing effect from the bulky carbonate side moiety and good comparison with residual reaction Enthalpy as evaluated from DSC (Figure S11). A similar trend is observed for the resin B samples, further emphasizing the lack of self-plasticization. Notably, however, no sample showed any trace of crystallinity. While this was expected and indeed observed in the rigid series, it was not ruled out in the soft series, owing to the presence of early crystallizable flexible methylene sequence in HDDA.^30^

Tensile and flexural properties were recorded, with results presented in Table 2 and summarized in Figure 4a. In the rigid mixture, the elastic modulus considerably decreases with high concentrations of GCI and incorporation of the biobased monomer. For instance, the elastic modulus drops by 34% from #A0 to #A3. However, elongation at break and tensile strength suggest that GCI toughens the formulations owing to the presence of a single difunctional double bond compared to the two (GDMA) and three (GPT) reactive functionalities in the other comonomers. This condition, however, may led to excessive linear propagation when GCI reaches 70 wt %, as observed in the differences in the mechanical behavior of the material. Elongation at break and tensile strength both increase from #A0 to #A2, with a 70% increase in #A2 compared to #A0, but decrease relative to other carbonate-containing formulations. A similar trend applies to the flexural properties, which are influenced differently by resin composition owing to the distinct type of stress applied. In tensile testing, the mechanical stress is applied parallel to the photocured layers, meaning that interlayer adhesion does not markedly contribute to tensile properties. In three-point flexural testing, both compressive and tensile stresses are applied perpendicular to the layer planes, making flexural properties closely related to interlayer adhesion. The hardness level remained constant at 88/89, with no profound difference observed in the amount of GCI. These data indicate a general toughening of the resin with the addition of GCI. Notably, the #A2 resin, with 63% biobased content, demonstrates excellent performance in elongation, doubling the stress at break. Soft mixtures exhibit a substantial increase in elastic modulus from #B0 to #B3 (+95%), likely owing to a decrease in the double cross-linker monomer (HDODA) in favor of the monofunctional comonomer (GCI). Conversely, elongation at break decreases from #B0 to #B3 (−93%). Tensile strength increases from #B0 to #B2 (+67%) and then levels off. Compared to rigid resins, these formulations exhibit more variability in their hardness, with values rising from 60 to 83 (#B0 to #B3), approaching those of the rigid resin. This increase can be attributed to the biobased GCI, which enhances hardness across the entire system. The impressive #B1 and #B2 formulations, with 53% and 65% biobased content, offer a remarkable combination of high tensile strength and good elongation at break (28.7% and 13.6%, respectively), making them suitable for applications beyond their intended use as soft resins.

**Table 2:** Mechanical Properties: Tensile, 3PB, and Hardness of the Eight Different Formulations Divided into Two Sets: Rigid (#AX) and Soft (#BX)^a^

#	elastic modulus (GPa)	elongation at break (%)	tensile strength (MPa)	flexural modulus (GPa)	deformation at break (%)	flexural strength (MPa)	hardness (shore D)	irradiation time (s/layer)
A0	1.25 ± 0.26	1.8 ± 0.8	22.6 ± 9.7	2.54 ± 0.17	3.2 ± 0.4	76.5 ± 6.4	89 ± 1	120
A1	1.05 ± 0.06	4.6 ± 1.4	41.7 ± 5.5	2.90 ± 0.25	2.6 ± 0.4	67.5 ± 9.2	89 ± 1	80
A2	0.97 ± 0.02	5.9 ± 0.7	38.5 ± 2.0	2.18 ± 0.10	2.9 ± 0.3	58.9 ± 2.5	89 ± 1	100
A3	0.82 ± 0.06	3.7 ± 1.0	31.1 ± 2.2	2.02 ± 0.05	2.6 ± 0.4	49.5 ± 2.8	88 ± 1	120
B0	0.05 ± 0.01	45.5 ± 2.9	9.2 ± 1.1	0.11 ± 0.01	14.1 ± 0.1	9.2 ± 0.1	60 ± 1	100
B1	0.33 ± 0.03	28.7 ± 3.4	15.7 ± 0.6	0.75 ± 0.08	12.0 ± 2.0	29.5 ± 2.8	78 ± 1	100
B2	0.83 ± 0.04	13.6 ± 2.5	27.7 ± 0.4	0.66 ± 0.05	5.4 ± 0.2	24.3 ± 1.5	82 ± 1	100
B3	1.06 ± 0.03	3.1 ± 0.5	28.3 ± 1.1	1.02 ± 0.05	5.1 ± 1.1	30.4 ± 2.2	83 ± 1	100

a For Each Set, Irradiation Time and the “Blank” (without the Biobased Monomer) are Implemented in #X0 Formulations.

In general, the use of GCI as a monomer, combined with two resins (rigid and soft), greatly impacts the final mechanical properties, resulting in a wide range of resin formulations with different features. As shown in Table 2, combining the GCI monomer with two types of resin yields different mechanical properties. In rigid resins, GCI does not notably alter the modulus, possibly owing to the intrinsic stiffness of the carbonate ring structure,^31^ making changes in elongation and strain more pronounced. By contrast, the monomer considerably affects the mechanical properties of soft resins. Increasing the concentration of the sample makes the sample progressively rigid and tough. Notably, the #B2 and #B3 resins exhibit specific behaviors characteristic of ductile polymers. These resins display an initial elastic modulus followed by plastic behavior, unlike the elastic nature of #B0 or the brittle response of #B3, likely owing to more pronounced linearity in the polymerized formulations. #B3 shows a brittle behavior, attributed to the presence of PEMA, which contributes to the softness of the polymer owing to its high free volume. The distinct mechanical behavior of #A1 compared with #A2 and #A3 may stem from the presence of substantial unreacted double bonds, as discussed in the DSC data. Additionally, two high-resolution objects, a wolf and a bracelet (Figure 4c), were printed using the most promising formulations (#A3 and #B2), both containing the highest GCI content (70 wt % GCI) and exhibited resolution retention typical of vat photopolymerization printers (Figure 4d/S14).

## Conclusions

This study highlights the successful synthesis and characterization of a novel 3D vat photopolymerization monomer, GCI, derived from renewable itaconic acid and glycerol. Characterization via NMR, IR, and ESI-MS confirmed the chemical structure of GCI, which demonstrated excellent compatibility with both rigid and soft resins at concentrations of up to 70 wt %, contributing to a high biobased content of up to 77 wt % in the overall formulations. All of the mechanical properties of the resin sets were tested. Generally, the single difunctional moiety and the free volume within GCI enhanced the elongation at break of rigid formulations, with #A2 doubling the stress at break compared to #A0, suggesting also an improved toughness (not measured). In soft resins, the mechanical behavior shifted toward increased rigidity, as evidenced by the #B2 formulation, which raised the tensile strength of the resins by 67%. The thermal analysis further indicated that the inclusion of GCI decreased the degradation temperature of the resins (high levels of thermal stability are nevertheless maintained) while enhancing their charring capability, imparting potential notable flame-retardant properties—an interesting feature for future studies.

Despite the well-known inertia of itaconate compared to fossil acrylic derivatives, the printing parameters were optimized, demonstrating that the high resolution typical of vat photopolymerization can be maintained with natural derivatives. These findings reveal the potential of GCI as a sustainable and versatile itaconic-based material for vat photopolymerization resins.

## Supplementary Material



## Notes

Additional experimental details, materials, methods, spectra, calculations, and photographs of the experimental setup (DOCX)
