# Supplementary material for: Vat Photopolymerization of Glycerol Carbonate Methyl Itaconate for Sustainable 3D Printing
Source: ACS Sustain Chem Eng. 2025 Jul 29;13(31):12421–9. doi: 10.1021/acssuschemeng.5c02799 (PMC12345386; doi:10.1021/acssuschemeng.5c02799)
Supplement: Supplementary file 1 [file sc5c02799_si_001.docx]

Vat Photopolymerization of Glycerol Carbonate Methylitaconate for Sustainable 3D-Printing

Rosario Carmenini^†^, Matteo Dalle Donne^†^, Erica Locatelli^†1^, Letizia Sambri^†^, Loris Giorgini^†^, Laura Mazzocchetti^†^ and Mauro Comes Franchini^†^*

^†^ Department of Industrial Chemistry “Toso Montanari”, University of Bologna, Bologna 40136, Italy.

**Supporting information**

**Contents**

[CHEMICAL CHARACTERIZATION S3](#_Toc203652390)

[*Glycerol carbonate [GC]* S3](#_Toc203652391)

[**Figure S1** ^13^C-NMR of glycerol carbonate (GC). S3](#_Toc203652392)

[**Figure S2** HSQC of glycerol carbonate (GC). S3](#_Toc203652393)

[**Figure S3** COSY of glycerol carbonate (GC). S4](#_Toc203652394)

[**Table S1** ^1^H/^13^C-NMR correlations of glycerol carbonate (GC). S4](#_Toc203652395)

[**Figure S4** ESI-MS of glycerol carbonate (GC). S5](#_Toc203652396)

[*Glycerol carbonate (methyl itaconate) [GCI]* S5](#_Toc203652397)

[**Figure S5** ^13^C-NMR of glycerol carbonate (methyl itaconate) (GCI). S5](#_Toc203652398)

[**Figure S6** HSQC of glycerol carbonate (methyl itaconate) (GCI). S6](#_Toc203652399)

[**Figure S7** COSY of glycerol carbonate (methyl itaconate) (GCI). S6](#_Toc203652400)

[**Table S2** ^1^H/^13^C-NMR correlations of glycerol carbonate (methyl itaconate) (GCI) S7](#_Toc203652401)

[**Figure S8** ESI-MS^+^ of glycerol carbonate(methyl itaconate) (GCI). S7](#_Toc203652402)

[MECHANICAL PROPERTIES S8](#_Toc203652403)

[***Table S3*** *Composition (wt.%) of the eight photocurable resins (including monomers and additives)* S8](#_Toc203652404)

[**Figure S9** Tensile stress-strain curves for the rigid (#A) and soft (#B) sets of resin. S8](#_Toc203652405)

[**Figure S10** 3PB stress-strain curves for the rigid (#A) and soft (#B) resin sets. S9](#_Toc203652406)

[THERMAL PROPERTIES S10](#_Toc203652407)

[**Figure S11** DSC of the eight resins sets. S10](#_Toc203652408)

[**Figure S12** Rheological analysis of the GCI monomer as a function of temperature at constant shear rate (1 Hz) [right]. S11](#_Toc203652409)

[**Figure S13** TGA analysis of all eight resin sets in air and nitrogen S11](#_Toc203652410)

[***Figure S14*** *Evaluation of the printing performances of resins* S12](#_Toc203652411)

[***Figure S15*** *Optical microscopic analysis* S13](#_Toc203652412)

[***Figure S16*** *Spectroscopic characterisation of the printing samples.* S13](#_Toc203652413)

[ENVIRONMENTAL METRIC CALCULATION S14](#_Toc203652414)

[*E-factor calculation:* S14](#_Toc203652415)

[***Scheme 1*** *Schematic representation of all the synthetical steps integrating all the molecular weight and the used masses.* S14](#_Toc203652416)

[*Atom Economy (AE) calculation:* S15](#_Toc203652417)

[*EcoScale calculation:* S15](#_Toc203652418)

[CHEMICAL HAZARDOUS S16](#_Toc203652419)

[Chemical hazardous during the chlorination step S16](#_Toc203652420)

[Eperimental procedure_Glycerol Carbonate/Monomethyl-itaconate: S17](#_Toc203652421)

[***Table S4*** *Reaction conditions and outcomes of the two reactions using glycerol carbonate and monomethyl itaconate directly, without the oxalyl chloride.* S17](#_Toc203652422)

[SOLVENT DEGRADATION TEST S18](#_Toc203652423)

[***Table S5*** *Specimens’ weight loss in four different solvents during the 30-day test.* S18](#_Toc203652424)

# **CHEMICAL CHARACTERIZATION**

## ***Glycerol carbonate [GC]***


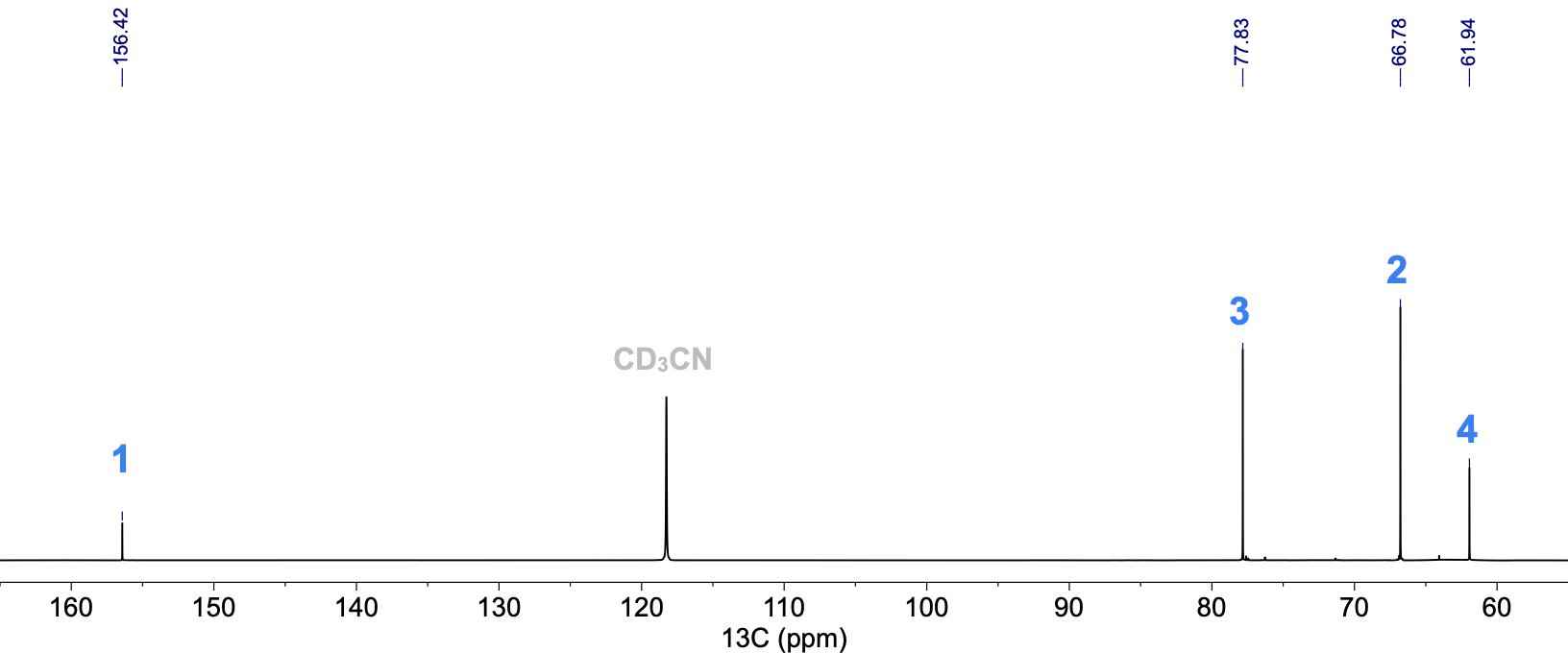


**Figure S1** ^13^C-NMR of glycerol carbonate (GC).

**Figure S2** HSQC of glycerol carbonate (GC).

**Figure S3** COSY of glycerol carbonate (GC).

**Table S1** ^1^H/^13^C-NMR correlations of glycerol carbonate (GC).

| **** | **^1^H (ppm)** | **^13^C (ppm)** |
| --- | --- | --- |
| **1** | -- | 156.42 |
| **2** | 4.30 and 4.47 | 66.78 |
| **3** | 4.75 | 77.83 |
| **4** | 3.79 and 3.76 | 61.94 |
| **5** | 3.32 | -- |


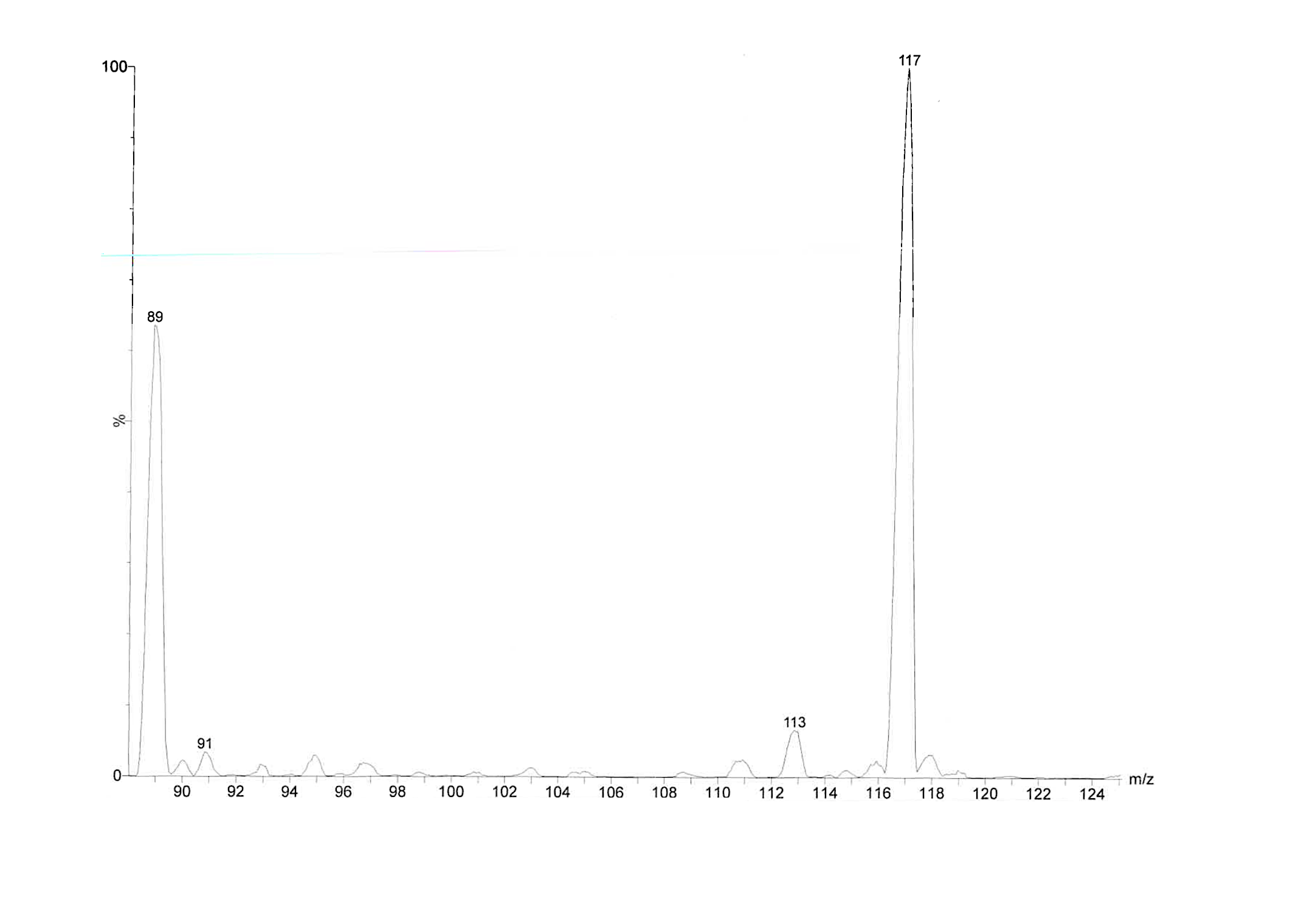


**Figure S4** ESI-MS of glycerol carbonate (GC).

## ***Glycerol carbonate (methyl itaconate) [GCI]***

**
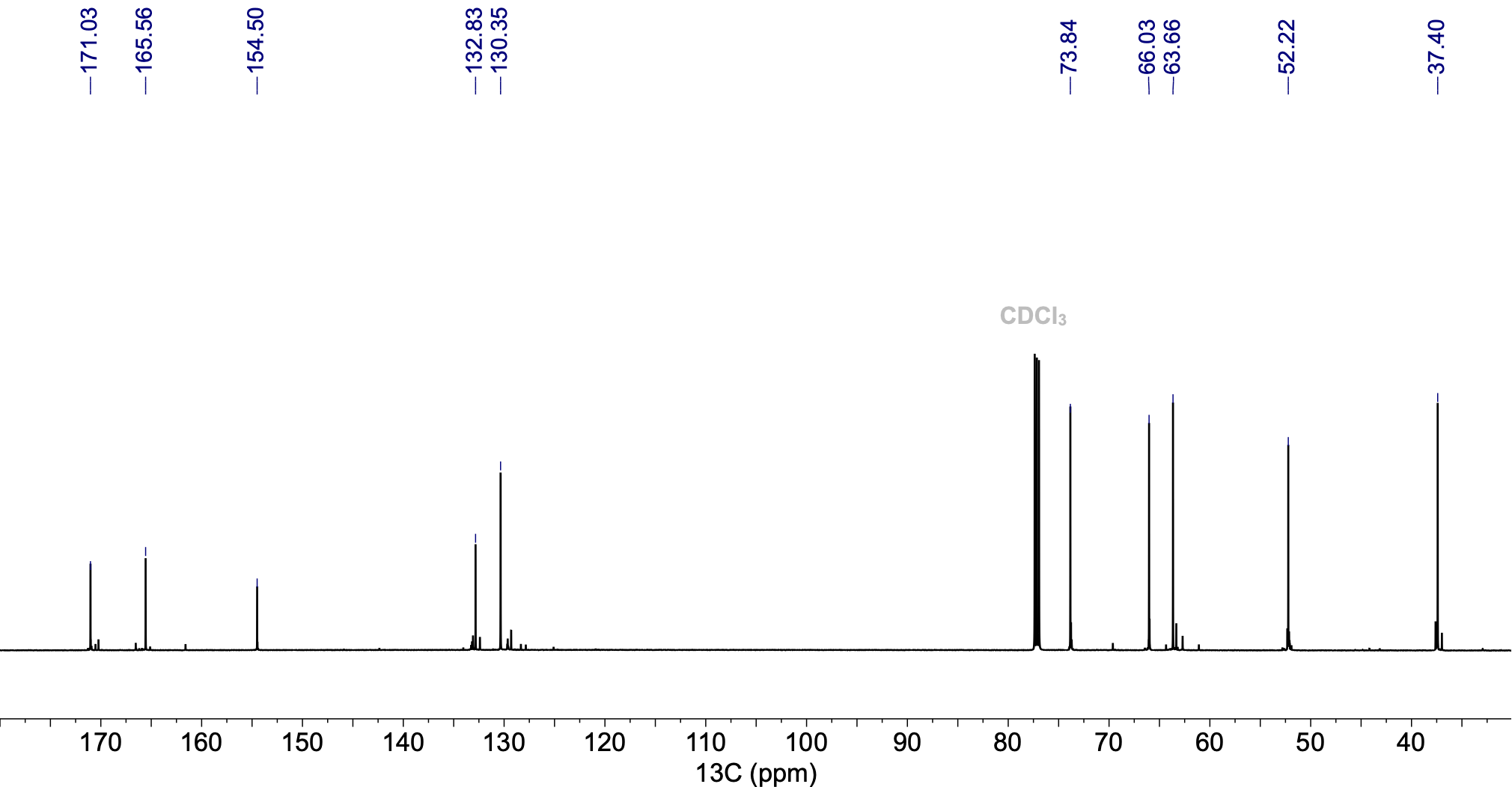
**

**Figure S5** ^13^C-NMR of glycerol carbonate (methyl itaconate) (GCI).

**Figure S6** HSQC of glycerol carbonate (methyl itaconate) (GCI).

**Figure S7** COSY of glycerol carbonate (methyl itaconate) (GCI).

**Table S2** ^1^H/^13^C-NMR correlations of glycerol carbonate (methyl itaconate) (GCI)

|  | **^1^H (ppm)** | **^13^C (ppm)** |
| --- | --- | --- |
| **1** | -- | 154.50 |
| **2** | 4.56 and 4.32 | 66.03 |
| **3** | 4.96 | 73.84 |
| **4** | 4.37 | 63.66 |
| **5** | -- | 165.56 |
| **6** | -- | 132.83 |
| **7** | 6.36 and 5.78 | 130.35 |
| **8** | 3.35 | 37.40 |
| **9** | -- | 171.03 |
| **10** | 3.69 | 52.22 |


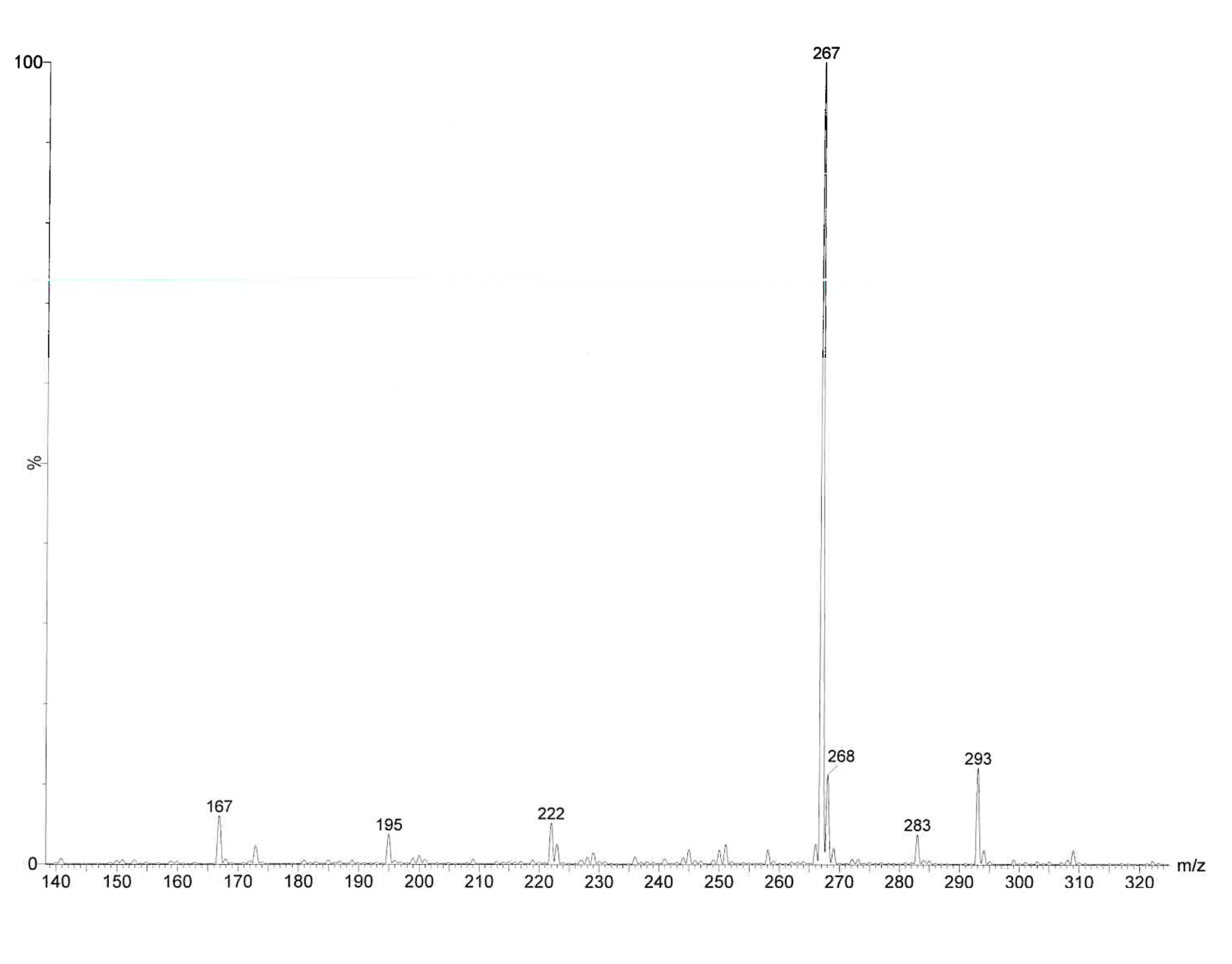


**Figure S8** ESI-MS^+^ of glycerol carbonate(methyl itaconate) (GCI).

# **MECHANICAL PROPERTIES**

| # | GCI | GPT | GDMA | PEMA | HDODA | Biobased content |
| --- | --- | --- | --- | --- | --- | --- |
| A0 | -- | 50 % | 50 % | -- | -- | 29 % |
| A1 | 30 % | 35 % | 35 % | -- | -- | 50 % |
| A2 | 50 % | 25 % | 25 % | -- | -- | 63 % |
| A3 | 70 % | 15 % | 15 % | -- | -- | 76 % |
| B0 | -- | -- | -- | 70 % | 30 % | 33 % |
| B1 | 30 % | -- | -- | 49 % | 21 % | 53 % |
| B2 | 50 % | -- | -- | 35 % | 15 % | 65 % |
| B3 | 70 % | -- | -- | 21 % | 9 % | 77 % |

***Table S3*** *Composition (wt.%) of the eight photocurable resins (for each formulation has been added another 10 wt.% including the photoinitiating system and plasticiser) and the corresponding overall biobased content (including monomers and additives).*

**Figure S9** Tensile stress-strain curves for the rigid (#A) and soft (#B) sets of resin.

**Figure S10** 3PB stress-strain curves for the rigid (#A) and soft (#B) resin sets.

# **THERMAL PROPERTIES**

**Figure S11** DSC of the eight resins sets.

**Figure S12** Rheological analysis of the GCI monomer as a function of temperature at constant shear rate (1 Hz) [right].

**Figure S13** TGA analysis of all eight resin sets in air (up) and nitrogen (down). All experiments were conducted from 25 °C to 700 °C.


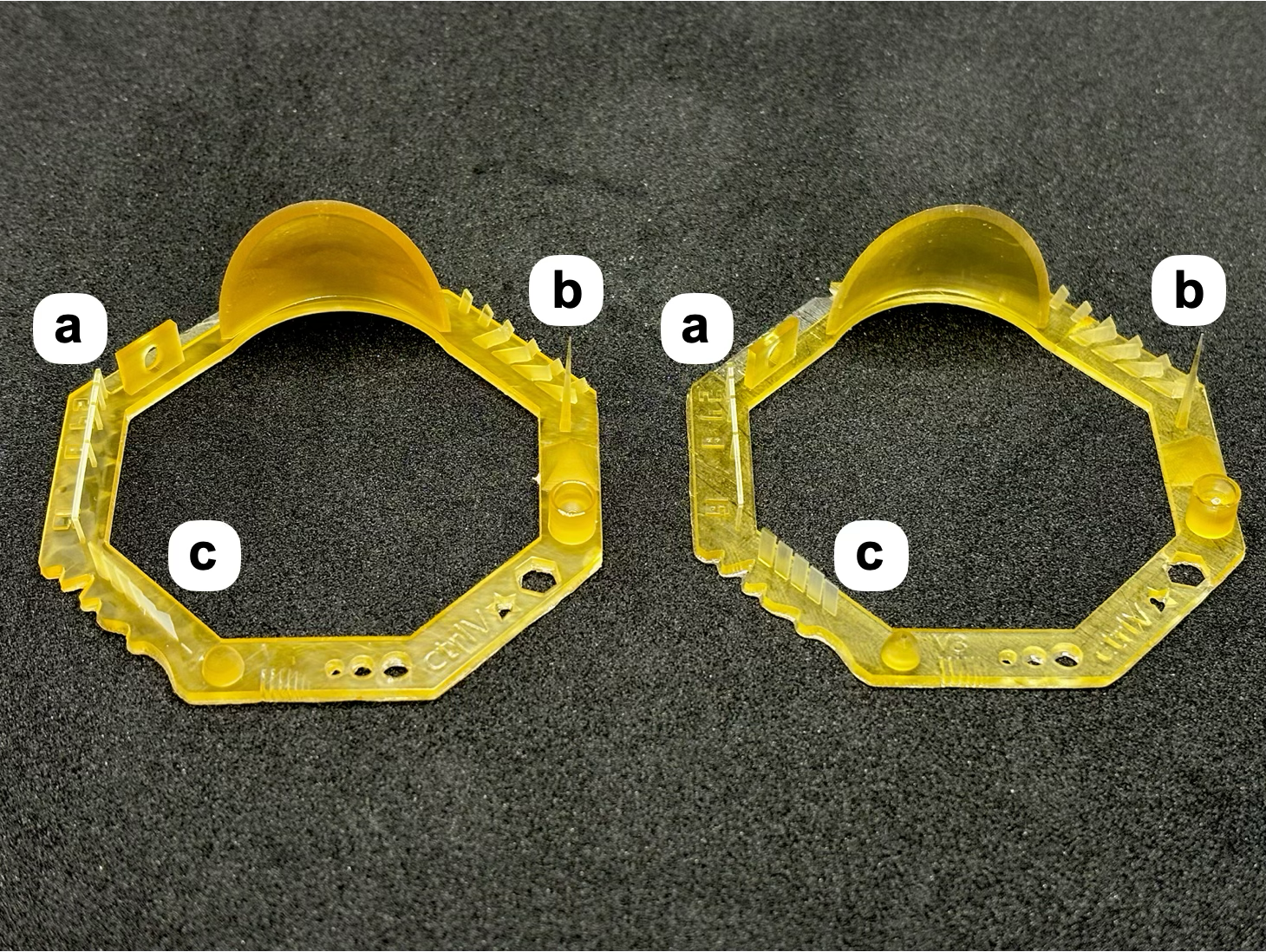


### ***Figure S14*** *Evaluation of the printing performances of resins #A3 (left) and #B3 (right).(a) Vertical hole in wall and bridge print with bridge length up to 16 mm; (b) spike and overhanging walls up to 70° inclination and (c)* *walls of decreasing thickness up to 0.1 mm.*


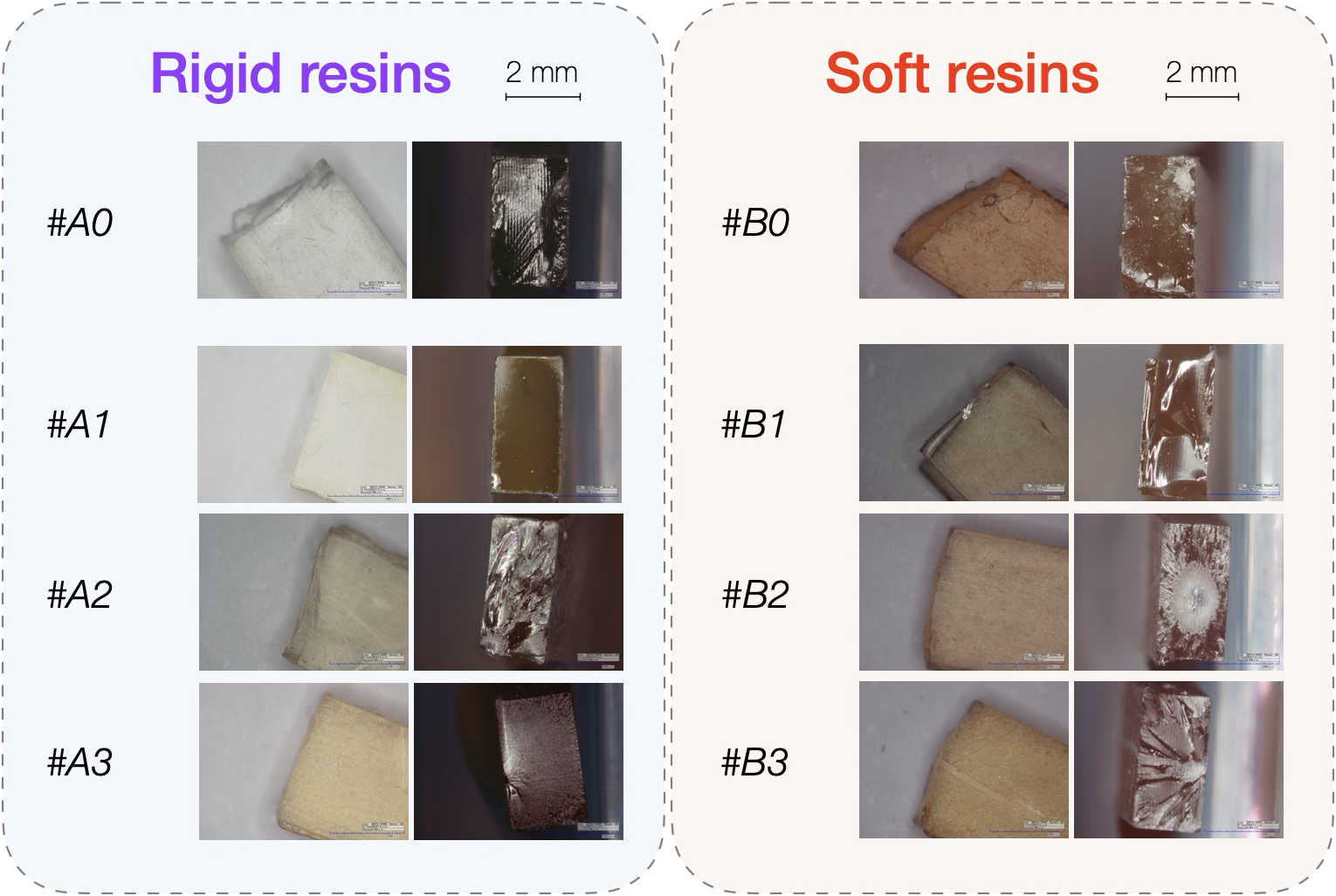


### ***Figure S15*** *Optical microscopic analysis (X50) of the fractured tensile test samples and the cross-section.*


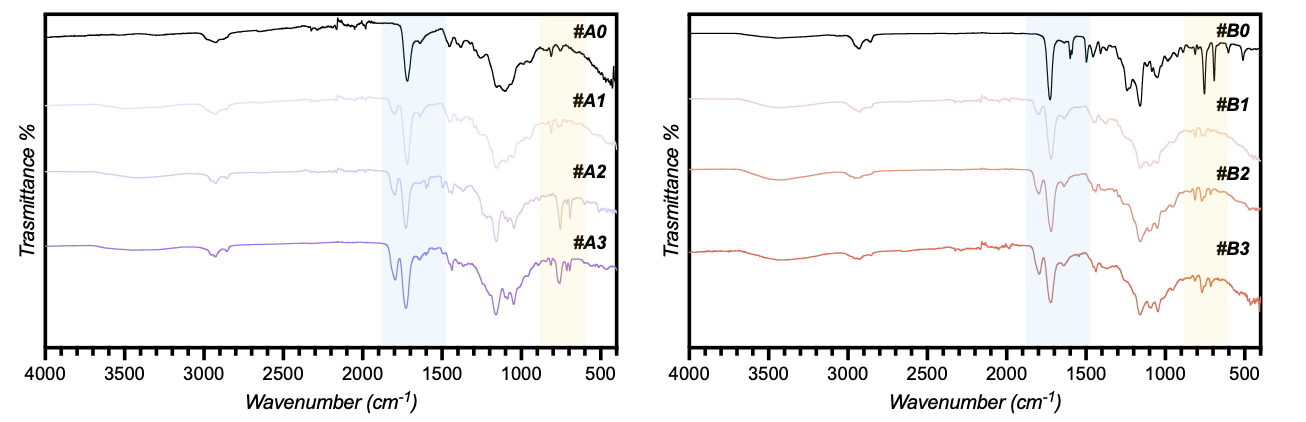


***Figure S16*** *Spectroscopic characterisation of the printing samples with all the #AX and #BX formulations with characteristic regions highlighted.*

# **ENVIRONMENTAL METRIC CALCULATION**

## ***E-factor calculation:***

### ***Scheme 1*** *Schematic representation of all the synthetical steps integrating all the molecular weight and the used masses.*

$$E_{factor}=\frac{{Kg}_{waste}}{{Kg}_{product}}$$

$${E_{factor}}_{TOT}={E_{factor}}_{R1}+ {E_{factor}}_{R2}+ {E_{factor}}_{R3}$$

$${E_{factor}}_{R1}=\frac{\left( 116+121+5,45 \right)-130}{130}=0,87$$

$${E_{factor}}_{R2}=\frac{\left( 216+121 \right)-209}{209}=0,61$$

$${E_{factor}}_{R3}=\frac{\left( 31+50 \right)-63}{63}=0,29$$

${E_{factor}}_{TOT}=0,87+ 0,61+ 0,29=1.77$

## ***Atom Economy (AE) calculation:***

$$AE=\frac{{MW}_{product} x 100}{{\Sigma MW}_{Raw Materials}+ {\Sigma MW}_{Reagents}}$$

$$AE=\frac{244,20}{92,09+60,06+114,13+126,93}=62 \%$$

***Process Mass Intensity (PMI) calculation:***

$$PMI=\frac{{mass}_{reactants}+{mass}_{reagents}{+ mass}_{catalyst}+ {mass}_{solvents}}{mass of isolated product}$$

Using the calculator available on the website (https://acsgcipr.org/tools/process-mass-intensity/) results in the calculation of the individual steps as:

- GC production: 1,9
- MonoMEIC production: 2,3
- GCI production: 4,0

The total PMI (Process Mass Intensity) is reported as 5.6, which aligns with the standard values observed in industrial processes, typically ranging from 1 to 5. Additionally, the importance of solvents in the potential scale-up process cannot be overstated, as their usage will be reduced in this scenario.

## ***EcoScale calculation:***

Following the Van Akenn et Al. (<https://ecoscale.cheminfo.org/calculator>) has been calculated.

The tool uses a scale from 0 to 100, where 0 indicates a completely failed reaction (0% yield) and 100 indicates an ideal reaction. It considers the energy used in the reaction as well as downstream purification processes.

The reaction conditions employed to produce a high-purity (> 98%) product are ranked on a scale from 0 to 100 as follows: > **75, excellent**; > **50, acceptable**; and < **50, inadequate**.

For the three different reaction steps, the EcoScale values are:

- **46.5** for the Glycerol Carbonate formation reaction, which is low due to the yield of 55%, as noted in the manuscript and which significantly impacts the final score;
- **82** for the formation of the monomethyl itaconoyl chloride;
- **88** for the GCI monomer formation reaction.

Overall, the entire process has an average EcoScale value of **72.2**, close to the threshold of 75, which the study’s authors classify as an **excellent value**.

# **CHEMICAL HAZARDOUS**

## **Chemical hazardous during the chlorination step**

“Potential safety issues using the Oxalyl chloride reagent indeed is a hazardous chemical that requires careful handling due to its corrosive and toxic nature. It can cause severe burns and damage to skin, eyes, and respiratory system. Exposure to oxalyl chloride can also lead to systemic effects like liver and kidney damage. It reacts violently with water, releasing flammable and toxic gases. Proper personal protective equipment (PPE), ventilation, and storage practices are crucial when working with oxalyl chloride.”

(<https://www.sigmaaldrich.com/IT/en/sds/aldrich/221015?userType=anonymous>)

The impurities resulting from the chlorination reaction are been:

- Removed by adding excess TEA during the reaction phase (to neutralise the developed HCl)

- Treated with water, extracting all the TEA salts on one side and the ‘clean’ product on the other. In this step, all chlorinated species (if any still exist) react with the water. In addition, possible traces of VOC are completely eliminated through the passage in a high vacuum pump. This results in a high purity of the final monomer, also according to the analysis evidence.

## **Eperimental procedure_Glycerol Carbonate/Monomethyl-itaconate:**

In a 100 mL round bottomed flask, monomethyl itaconate (14,4 g, 0,1 mol) and glycerol carbonate (11, 81 g, 1.5 mol) are mixed with (5 mmol) of catalyst (see table S4). The mixture is heated to 150°C for 1 h during which the methanol produced by the transesterification reaction is removed by distillation. At the end of the reaction, the mixture is cooled to room temperature and extracted with EtOAc/water for 3 times. The organic phase is therefore dried over sodium sulfate and evaporated as a pale yellow liquid. Yield: 30%.

***Table S4*** *Reaction conditions and outcomes of the two reactions using glycerol carbonate and monomethyl itaconate directly, without the oxalyl chloride.*

| # | Catalyst | Solvent | Reaction conditions | Reaction outcome |
| --- | --- | --- | --- | --- |
| 1 | ZnCl_2_ | Neat | 150°C/1h | Brown viscous liquid, presence of byproducts |
| 2 | DBTO | Neat | 150°C/1h | Yellow viscous liquid with low yield |

# **SOLVENT DEGRADATION TEST**

The solvent degradation test has been performed selected four different solvents: water, isopropyl alcohol, ethyl acetate, and acetone putting the tensile specimens in vials (a duplicate was made for each test). Additionally, two more objects were moulded with the GCI-based resin, alongside the objects moulded with the Formlabs Elastic and Formlabs Tough resins, for comparison with commercial resins.

Below, we report the results of the 30-day test, measuring the mass loss of the test specimens over time, each week.

***Table S5*** *Specimens’ weight loss in four different solvents during the 30-day test.*

|  | Weight loss (%wt.) | | | | | | | | | | |
| --- | --- | --- | --- | --- | --- | --- | --- | --- | --- | --- | --- |
| *#* | ***A0*** | ***A1*** | ***A2*** | ***A3*** | ***Fomlabs TOUGH*** | ***Formlabs FLEXIBLE*** | ***B0*** | ***B1*** | ***B2*** | ***B3*** |  |
| ***Water*** | +4,7 | +2,7 | +1,0 | +2,1 | +3,0 | +0,4 | -0,08 | +3,7 | +2,9 | +2,4 |  |
| ***iPrOH*** | -1,2 | -1,1 | \ | -0,2 | +29,6 | +26,6 | + 2,5 | -0,7 | -1,1 | -1,2 |  |
| ***EtOAc*** | -0,3 | +4,2 | \ | -5,7 | +31,3 | +72,5 | +1,3 | +2,9 | +1,7 | -5,7 |  |
| ***Acetone*** | +0,4 | -2,3 | \ | \ | +23,9 | + 41,6 | -19,43 | -1,5 | -2,8 | -13,0 |  |

These tests reveal that specimens belonging to series #A2 and #A3 exhibit reduced strength after four weeks in organic solvents, leading to disintegration. Generally, all the samples show an increase in weight in water and a significant decrease in weight in more polar solvents, such as acetone and ethyl acetate, while in isopropanol the losses are limited. Commercial resins behave in the opposite way to laboratory formulations by absorbing a lot of solvent and swelling significantly, even in iPrOH.
